# Allosteric modulation of G protein-coupled receptor signaling

**DOI:** 10.3389/fendo.2023.1137604

**Published:** 2023-02-16

**Authors:** Siyuan Shen, Chang Zhao, Chao Wu, Suyue Sun, Ziyan Li, Wei Yan, Zhenhua Shao

**Affiliations:** Division of Nephrology and Kidney Research Institute, State Key Laboratory of Biotherapy, West China Hospital, Sichuan University, Chengdu, Sichuan, China

**Keywords:** G protein-coupled receptors (GPCRs), allosteric modulator, structural investigations, GPCR signaling regulation, allosteric drug discovery

## Abstract

G protein-coupled receptors (GPCRs), the largest family of transmembrane proteins, regulate a wide array of physiological processes in response to extracellular signals. Although these receptors have proven to be the most successful class of drug targets, their complicated signal transduction pathways (including different effector G proteins and β-arrestins) and mediation by orthosteric ligands often cause difficulties for drug development, such as on- or off-target effects. Interestingly, identification of ligands that engage allosteric binding sites, which are different from classic orthosteric sites, can promote pathway-specific effects in cooperation with orthosteric ligands. Such pharmacological properties of allosteric modulators offer new strategies to design safer GPCR-targeted therapeutics for various diseases. Here, we explore recent structural studies of GPCRs bound to allosteric modulators. Our inspection of all GPCR families reveals recognition mechanisms of allosteric regulation. More importantly, this review highlights the diversity of allosteric sites and presents how allosteric modulators control specific GPCR pathways to provide opportunities for the development of new valuable agents.

## Introduction

G protein-coupled receptors (GPCRs), the most successful class of drug targets, regulate almost all physiological responses by sensing diverse external signals, including light, hormones, ions, and proteins (1–3). GPCRs share a typical architecture with seven transmembrane helices and exhibit conformational dynamics under physiological conditions (4–7). Historically, most marketed pharmaceuticals target orthosteric sites on GPCRs, where endogenous signal molecules are bound, to control conformational changes and regulate signal transduction (8). However, the highly conserved property of orthosteric sites among GPCR subtypes and their complicated signaling pathways cause numerous difficulties for the development of specific and safe therapeutics. Unlike classical orthosteric ligands, allosteric modulators bind to a distinct site on the receptor. Upon binding, allosteric modulators can remotely regulate the conformational transition of GPCRs and specifically regulate their signal transduction pathways, offering new strategies for the development of GPCR-targeted drugs (8–10).

Emerging allosteric modulators of GPCRs are chemically diverse (including proteins, peptides, small molecules, ions, and lipids) but can be divided into three categories according to their pharmacological properties on receptor signaling (11, 12): (i) positive allosteric modulators (PAMs) work synergistically with orthosteric agonists to enhance downstream signals; (ii) negative allosteric modulators (NAMs) modulate the affinity of orthosteric ligands to a receptor, ultimately downregulating or blocking orthosteric agonism; and (iii) neutral allosteric modulators do not have a positive or negative regulatory effect on signal transduction of the receptor after binding to the allosteric site. Some allosteric modulators also exhibit intrinsic agonism (known as ago-PAM) or inverse agonist profiles when used alone (13, 14). Notably, such modulator-mediated allostery depends on orthosteric ligands and receptor signaling pathways, and is therefore deemed to be probe-dependent (14–16). For example, the NTSR1 modulator SBI-55 was found a PAM for β-arrestin recruitment but a NAM-agonist at G protein pathway when cooperating with the orthosteric ligand neurotensin, thus allosterically induced biased signaling (17–19).

According to the Allosteric Database (ASD, http://mdl.shsmu.edu.cn/ASD) (20), four allosteric drugs targeting GPCRs (Cinacalcet, Ticagrelor, Ivermectin, and ATx-201) have been approved by the U.S. Food and Drug Administration (FDA), and another 25 are in clinical trials (**Table 1**). Among agents currently on the market, Cinacalcet and ATx-201 positively modulate the Gq signaling of extracellular Ca^2+^-sensing receptor (CaSR) and neuropeptide Y receptor type 4 (NPY_4_R), respectively (21, 26), while Ticagrelor negatively regulates the Gi pathway of P2Y receptor 12 (26). Avacopan was recently approved for antineutrophil cytoplasmic antibody-associated vasculitis (52) but has not been updated in the ASD database. Avacopan is a NAM of C5a anaphylatoxin chemotactic receptor 1 that inhibits both Gi protein and β-arrestin signals, which may confer signal bias (29). With recent breakthroughs in structural biology, more abundant allosteric sites and regulatory mechanisms of GPCRs have been identified, providing a basis for accelerating the development of allosteric drugs. This mini-review summarizes recent structural investigations of allosteric regulation of Class A, Class B, and Class C GPCRs (**Table 2**) and exemplars of allosteric modulator-bound GPCR structures to provide insight into the allosteric mechanisms of GPCR transduction signaling.

**Table 1:** List of allosteric drugs target GPCR in clinical trials.

Allosteric drugs	Condition	GPCR Target	Data	Action	Signaling	References
Approved						
Cinacalcet	Hyperparathyroidism	CasR	April 2002	PAM	Ca^2+^ mobilization	(21–25)
Ticagrelor	Stroke; Acute coronary syndrome	P2Y_12_	July 2011	NAM	Gi	(26)
Ivermectin	Parasitic roundworm infections	GABA_B_	1987	PAM	Unclear	(27)
ATx-201	viral and bacterial infections; Atopic dermatitis; Cancer; Rheumatoid arthritis;	NPY4	2019	PAM	Gq	(28)
Avacopan	ANCA-Associated Vasculitis	C5aR1	October 2021	NAM	Gi/β-arrestin2	(29)
Phase III						
Vercirnon	Inflammatory bowel disease	CCR9	September 2017 (completed)	NAM	Ca^2+^ mobilization	(30)
BMS-986165	Plaque psoriasis; Psoriatic arthritis; Crohn’s disease; Systemic lupus erythematosus	mGluR4	January 2023 (Recruiting)	Unclear	Unclear	ASD database
mavoglurant	Fragile X syndrome	mGluR5	March 2016 (Terminated)	NAM	Gq	(31)
ADX-48621	Parkinson’s disease levodopa- induced dyskinesia	mGluR5	April 2022 (Recruiting)	NAM	Gq	(32)
Basimglurant	Fragile X syndrome	mGluR5	December 2022 (Recruiting)	NAM	Gq	(33)
Phase II						
ADX-10059	Gastroesophageal reflux; Migraines	mGluR5	July 16, 2012 (completed)	NAM	Unclear	(34)
T-62	Neuropathic pain; Postherpetic neuralgia (PHN)	A_1_AR	June 8, 2012 (Terminated)	PAM	Unclear	(35)
AZD-8529	Smoking cessation therapy; Schizophrenia	mGluR2	November 2017 (completed)	PAM	Gi	(36)
ADX-71149	Epilepsy; Anxiety disorder; Schizophrenia	mGluR2	January 2023(Recruiting)	PAM	Gi	(37, 38)
MK-7622	Pain; Schizophrenia; Sleep disorder; Dementia, Alzheimer’s type	M_1_R	September 2018 (terminated)	PAM	Gq	(39–41)
LY-3154207	Dementia, Parkinson	DRD1	July 23, 2021(completed)	PAM	Gs	(42, 43)
ASP-4345	Schizophrenia; Cognitive disorders	DRD1	May 2022 (completed)	PAM	Unclear	(44)
PXT-002331	Parkinson’s disease	mGluR4	March 2020 (completed)	PAM	Gi	(45)
ASP-8302	Detrusor underactivity (Underactive bladder)	M_3_R	July 2022 (completed)	PAM	Gq	(46)
Emraclidine	Schizophrenia	M_4_R	December 2022 (Recruiting)	PAM	Gi	(47)
Phase I						
HTL-0014242	Neurological disorders; Psychiatric disorders	mGluR5	April 2021 (completed)	NAM	Gq	(48)
^[11C]^JNJ-4229193	Diagnostics	mGluR2	Unclear	PAM	Gi	(49)
JNJ-2463	Non-alcoholic steatohepatitis; Nephropathy, diabetic; Non-alcoholic fatty liver disease (NAFLD); Fibrosis; Metabolic Diseases	CB1	Unclear	NAM	Unclear	https://profiles.biocentury.com/products/namacizumab_(jnj-2463_ryi-018)
RG-7342	Schizophrenia	mGluR5	Unclear	PAM	Unclear	ASD database
JNJ-55375515	Cognitive disorders; Psychosis	mGluR2	October 2018 (Completed)	NAM	Unclear	ASD database
ODM-106	Essential tremor	GABA_B_	December 2016 (Completed)	PAM	Unclear	ASD database
MK-6884	Dementia, Alzheimer’s type	M_4_R	September 2022 (completed)	PAM	Gq	(50)
RGH-618	Anxiety disorder	mGluR5	Unclear	NAM	Unclear	ASD database
TAK-071	Lewy body dementia; Neurological Disorders; Dementia, Alzheimer’s type	M_1_R	December 2022 (Active, not recruiting)	PAM	Gq	(51)
VU-319	Pain; Sleep disorder; Dementia, Alzheimer’s type	M_1_R	February 2020 (completed)	PAM	Unclear	ASD database

**Table 2:** Recent structures of GPCRs in complex with allosteric modulators.

Allosteric modulators	GPCR Target	Action	Binding site	Signaling	PDB code	References
Class A						
Cmpd-6FA	β2AR	PAM	Within 7TMD near TM2/TM3/TM4 and ICL2	Gs	6N48	(53)
Cmpd-15PA	β2AR	NAM	Intracellular ends of TM1, TM2, TM6, TM7, helix 8, and ICL1	Gs	5X7D	(54)
AS408	β2AR	NAM	Outside 7TMD near TM3//TM5	Gs/β-arrestin	6OBA	(55)
AP8	GPR40	ago-PAM	Outside 7TMD near TM3/TM4/TM5 and ICL2	Gq	5TZY	(56)
MK-8666	GPR40	Partial agonist	Outside the 7TMD near TM3/TM4	Gq	5TZR	(56)
TAK-875	GPR40	Partial agonist	Outside the 7TMD near TM3/TM4	Gq	4PHU	(57)
LY3154207	DRD1	PAM	Outside 7TMD near TM3/TM4/TM5 and ICL2	Gs	7CKZ	(43, 58)
ZCZ011	CB1	PAM	Outside 7TMD near TM2/TM3/TM4	Gi	7FEE;7WV9	(59)
ORG27569	CB1	NAM	Outside 7TMD near TM2/TM3/TM4	Gi	6KQI	(60)
MIPS521	A_1_R	PAM	Outside 7TMD near TM6/TM7	Gi	7LD3	(61)
LY2116920	M_2_R	PAM	Top of extracellular vestibule	GoA/β-arrestin	4MQT;7T94;7T96	(62, 63)
2-PCCA	GPR88	PAM	Outside 7TMD near the cytoplasmic ends of TM5/TM6	Gi	7EJK	(64)
ML382	MRGPRX_1_	PAM	Within 7TMD near TM1/TM2/TM3/TM6/TM7	Gq	8DWG	(65)
BPTU	P2Y_1_R	antagonist	Outside 7TMD near TM1/TM2/TM3	unclear	4XNV	(66)
AZ3451	PAR2	antagonist	Outside 7TMD near TM2/TM3/TM4	Gq/β-arrestin	5NDZ	(67)
AZ8838	PAR2	antagonist	Extracellular vestibule near TM1/TM2/TM3/TM7	Gq/β-arrestin	5NDD	(67)
CCX168	C5aR1	antagonist	Outside 7TMD near TM3/TM4/TM5	Gi/β-arrestin2	6C1R	(68)
NDT9513727	C5aR1	antagonist	Outside 7TMD near TM3/TM4/TM5	Gi/β-arrestin2	5O9H	(69)
CCR2-RA-[R]	CCR2	antagonist	Intracelluar surface near TM6/TM7/H8	Gi	5T1A	(70)
vercirnon	CCR9	antagonist	Intracellular surface near TM6/TM7/H8	Gi	5LWE	(71)
Class B						
PF-06372222	GLP-1R	NAM	Outside 7TMD near TM5/TM6/TM7	Gs	5VEW	(72)
NNC0640	GLP-1R	NAM	Outside 7TMD near TM5/TM6/TM7	Gs	5VEX	(72)
LSN3160440	GLP-1R	PAM	Within 7TMD near TM1/TM2	Gs	6VCB	(73)
compound 2	GLP-1R	ago-PAM	Outside 7TMD near TM6	Gs	7DUR; 7DUQ;7E14	(74)
NNC0640	GCGR	NAM	Outside 7TMD near TM5/TM6/TM7	Gs	5XEZ;5XF7	(75)
MK-0893	GCGR	NAM	Outside 7TMD near TM5/TM6/TM7	Gs	5EE7	(76)
CP-376395	CRF_1_R	NAM	Within 7TMD near TM3/TM5/TM7	Gs	4K5Y	(77)
Class C						
GS39783	GABA_B_	PAM	Intracellular tips of TM6-mediated dimerization interface	Gi/o	6UO8	(78)
rac-BHFF	GABA_B_	PAM	Intracellular tips of TM5-TM6 of GB1 and TM6 of GB2	Gi/o	7CA3;7C7Q;7EB2	(79–81)
Evocalcet	CaSR	PAM	Outside 7TMD near TM2/TM5/TM6/TM7	Gq	7DD7	(82, 83)
NPS-2143	CaSR	NAM	Outside 7TMD near TM3/TM5/TM6/TM7	Gq	7DD5;7SIN;7M3J	(82–84)
R-568	CaSR	PAM	Outside 7TMD near TM2/TM5/TM6/TM7	Gq	7SIL	(84)
Cinacalcet	CaSR	PAM	Outside 7TMD near TM2/TM5/TM6/TM7	Gq	7M3F	(83)
Etelcalcetide	CaSR	PAM	At the LB2 interface(ECD)	Gq	7M3G	(83)
FITM	mGluR1	NAM	Outside 7TMD near TM2/TM3/TM4/TM5 and ECL2	Unclear	4OR2	(85)
JNJ-40411813	mGluR2	PAM	Outside 7TMD near TM3/TM5/TM6/TM7	Gi	7E9G	(38)
NAM563	mGluR2	NAM	Outside 7TMD near TM3/TM5/TM6/TM7	Gi	7EPE	(86)
NAM597	mGluR2	NAM	Outside 7TMD near TM3/TM5/TM6/TM7	Gi	7EPF	(86)
VU6001966	mGluR2	NAM	Unclear	Gi	7MTQ	(87)
ADX55164	mGluR2	ago-PAM	Outside 7TMD near TM3/TM5/TM6	Gi	7MTR	(87)
VU0650786	mGluR3	NAM	Outside 7TMD near TM3/TM5/TM6/TM7	Gi	7WI6	(88)
Mavoglurant	mGluR5	NAM	Outside 7TMD near TM2/TM3/TM5/TM6/TM7	Gq	4OO9	(89)
Fenobam	mGluR5	NAM	Outside 7TMD near TM2/TM3/TM5/TM6/TM7	Gi	6FFH	(90)
M-MPEP	mGluR5	NAM	Outside 7TMD near TM2/TM3/TM5/TM6/TM7	Gi	6FFI	(90)
MMPIP	mGluR7	NAM	Unclear	Gi	7EPC	(86)

## Allosteric modulation mechanism of Class A GPCRs transduction

Class A GPCRs (also called rhodopsin-like GPCRs), including aminergic receptors, lipid receptors, peptide receptors, and other receptors, are the single most fruitful drug target (91–93). The first structure of rhodopsin was determined two decades ago (94), while the first GPCR signaling complex [β2 adrenergic receptor (β2AR)-Gs bound to an agonist] was reported in 2011 (95). Growing numbers of receptors in complex with orthosteric or allosteric ligands are being published, providing opportunities to understand conformational transitions of receptors upon activation and allosteric modulation.

**Figure 1:**
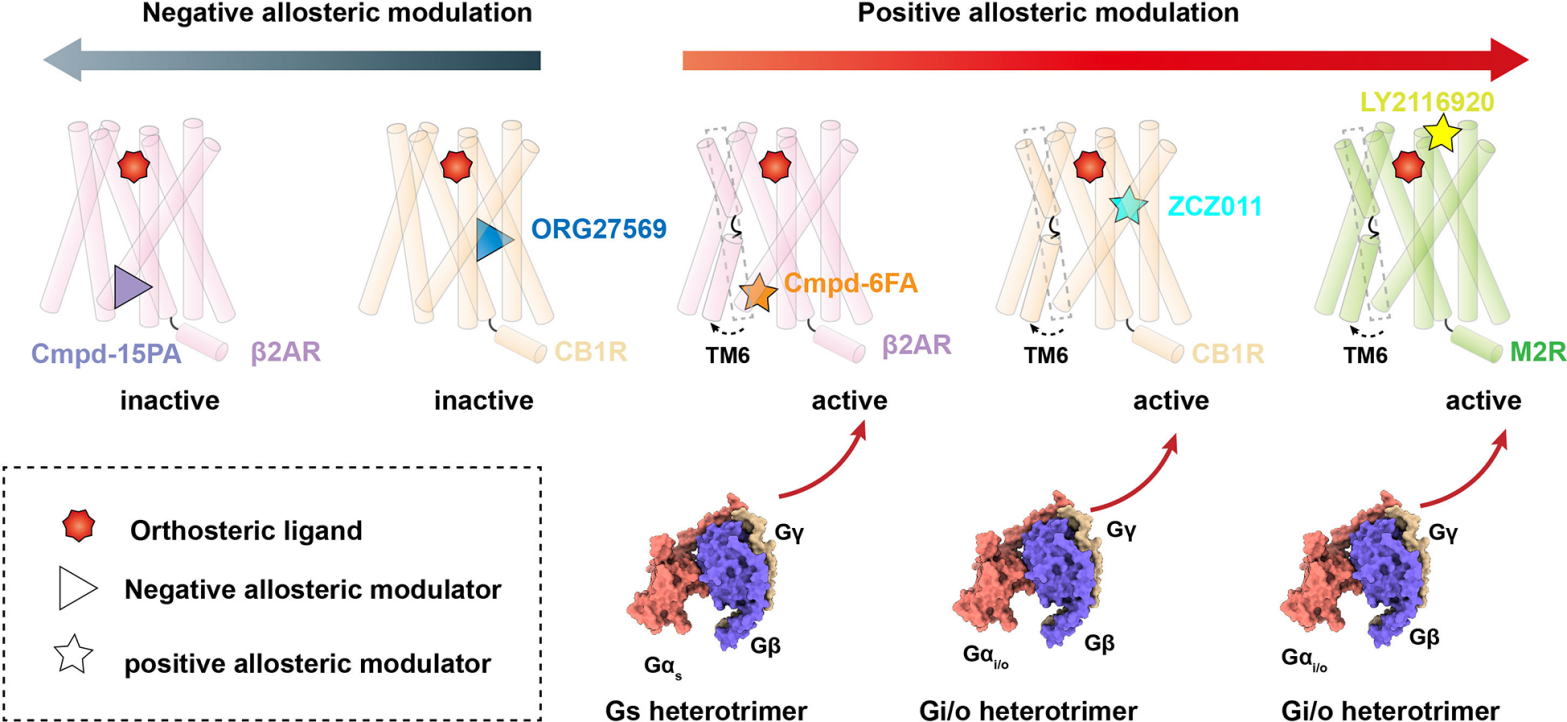
Allosteric modulation of Class A GPCRs. Negative allosteric modulation of β2AR and CB1R signaling transduction (left) and positive allosteric modulation of β2AR, CB1R and M2R signaling transduction (right).

**Figure 2:**
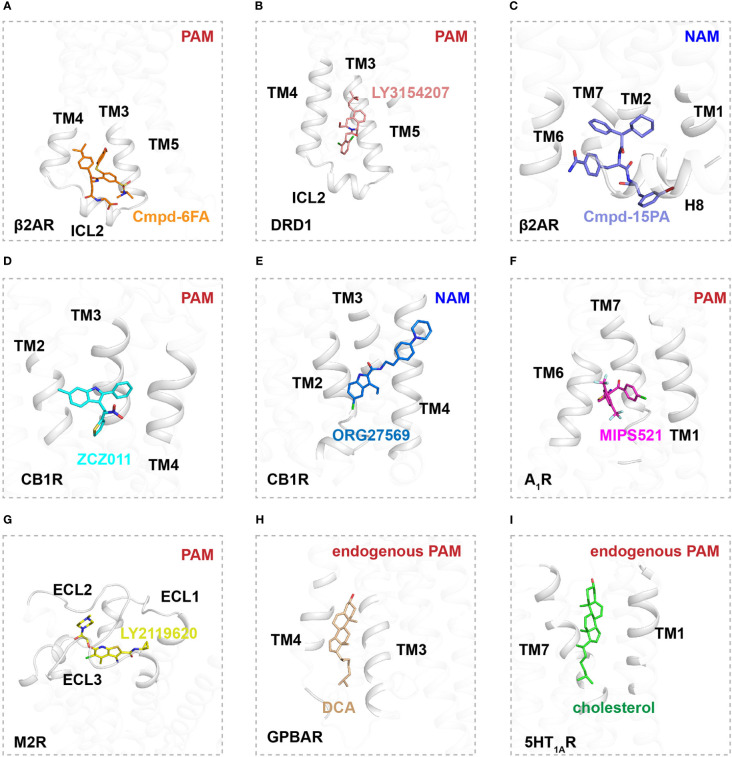
Binding sites of allosteric modulators in Class A GPCRs. **(A)** Interactions of Cmpd-6FA (orange, sticks) with TM2, TM3, TM4, and ICL2 of β2AR (gray, cartoon) (PDB ID: 6N48). **(B)** Interactions of LY3154207 (pink, sticks) with TM2, TM3, TM4, and ICL2 of DRD1 (gray, cartoon) (PDB ID: 7CKZ). **(C)** Interactions of Cmpd-15PA (purple, sticks) with TM1, TM2, TM6, TM7, H8 and ICL1 of β2AR (gray, cartoon) (PDB ID: 5X7D). **(D)** Interactions of ZCZ011 (blue, sticks) with TM2, TM3 and TM4 of CB1R (gray, cartoon) (PDB ID: 7WV9). **(E)** Interactions of ORG27569 (deep blue, sticks) with TM2, TM3 and TM4 of CB1R (gray, cartoon) (PDB ID: 6KQI). **(F)** Interactions of MIPS521 (magentas, sticks) with TM6 and TM7 of A1R (gray, cartoon) (PDB ID: 7LD3). **(G)** Interactions of LY2116920 (yellow, sticks) with extracellular region of M2R (gray, cartoon) (PDB ID: 4MQT). **(H)** Interactions of DCA (wheat, sticks) with extracellular region of GPBAR (gray, cartoon) (PDB ID: 7CFM). **(I)** Interactions of cholesterol (green, sticks) with TM1 and TM7 of 5-HT1A (gray, cartoon) (PDB ID: 7E2Y).

The β2AR is a well-characterized canonical receptor that exhibits dynamic conformational changes in membrane bilayers (96). Ligand Cmpd-6FA, identified as a PAM of β2AR, exhibits robust positive cooperativity with orthosteric agonists to activate Gs signaling. The structure of this receptor complex reveals that the binding pocket of Cmpd-6FA is formed by the intracellular regions of transmembrane helix 2 (TM2), TM3, TM4, and intracellular loop 2 (ICL2) (53)(**Figures 1**, **2A**). Upon Cmpd-6FA binding, ICL2 undergoes notable rearrangement from a disordered loop to a helical shape. This stabilization of ICL2 by Cmpd-6FA may explain its allosteric communication with agonists, e.g., enhanced binding affinity of orthosteric agonists (**Figures 1**, **2A**). Similar binding of allosteric modulators to the region above the ICL2 has been reported for other GPCRs, such as GPR40 with ago-PAM AP8(56) and DRD1 with PAM LY3154207 (43, 58) (**Figure 2B**). These findings indicate that certain PAM ligands can stabilize an intermediate receptor state and further activate intracellular effectors. Especially, 2-PCCA is a synthetic ago-allosteric modulator of orphan receptor GPR88, which has two binding sites. One is canonical orthosteric site formed by TM3, TM4, TM5, TM7 and ECL2, another is formed by the cytoplasmic ends of TM5 TM6 and C-terminus of the Gi1 α5 helix. 2-PCCA binding to GPR88 and directly interact with G protein, which stabilizes the active state of the receptor (64). In contrast, Cmpd-15PA, a negative allosteric modulator of β2AR, binds an intracellular allosteric site formed by the intracellular ends of TM1, TM2, TM6, TM7, helix 8, and ICL1 (**Figures 1**, **2C**). The structure of β2AR with Cmpd-15PA reveals that the NAM molecule restricts the inactive conformation of receptor by making direct contacts with residues N69^2.40^, I72^2.43^ and T274^6.36^, thereby decreasing its binding affinity for the agonist isoproterenol and activation of corresponding signaling (54, 97, 98).

Cannabinoid receptor 1 (CB1) is the most abundant GPCR in the central nervous system (CNS), whereby it regulates diverse physiological and pathological processes (99). Plant-derived cannabinoids and synthetic agonists are under clinical trials for treatment of various diseases (100, 101); unfortunately, some have undesirable side effects referred to as “cannabimimetic” effects (102–108). Allosteric modulators undoubtedly release the untapped potential of CB1 by cooperatively or non-cooperatively regulating the efficacy of its signal transduction with orthosteric ligands (109). ZCZ011, a PAM ligand of CB1, was recently reported to bind an extrahelical site in TM2, TM3, and TM4 (**Figures 1**, **2D**). Our molecular dynamics simulations indicate that ZCZ011 could increase the distribution of receptor conformations by promoting rearrangements of TM2 to enhance Gi protein-mediated signaling (59). Distinct from the PAM mechanism of ZCZ011 on CB1, the NAM ORG27569 was found to bind to the lower half of the TM2-TM3-TM4 surface (**Figures 1**, **2E**). Accordingly, the mechanism of allosteric antagonism might involve ORG27569 capturing an intermediate state of CB1 in which toggle-switch residues F^3.36^–W^6.48^ at the base of the agonist-binding pocket adopt an inactive conformation, thereby inhibiting Gi-protein activation of CB1 (59).

Allosteric sites in GPCRs are not conserved and exhibit divergent pharmacological properties, providing new therapeutic strategies for a wide array of diseases (13). In particular, MIPS521 was found to act as a PAM of adenosine 1 receptor (A_1_R) by binding a novel allosteric site formed by TM6 and TM7 (**Figure 2F**). In this interaction, the ligand exerts positive allosteric modulation cooperatively with endogenous adenosine to further stabilize the Gi-protein activation state of A_1_R (61). In addition to extrahelical and intracellular allosteric sites, allosteric modulators can bind to the extracellular region of receptors. LY2116920, a well-characterized ligand, acts as a PAM of muscarinic acetylcholine receptor M2 (**Figures 1**, **2G**) by binding a new allosteric site formed above the orthosteric pocket to synergistically modulate receptor signal transduction by preventing agonist dissociation from the orthosteric pocket (62, 63). Furthermore, TAK-875 was characterized as an ago-allosteric modulator of GPR40 and is under phase III clinical trials for the treatment of type-2 diabetes, further structural determination reveals that TAK-875 binds to a non-canonical site formed by TM3, TM4, TM5 and ECL2 (57).

Recently, an emerging class of GPCR allosteric modulators, which exert pathway-specific effects on receptor signaling, are defined as biased allosteric modulators (BAMs). For instance, SBI-553 was reported as an arrestin-biased PAM of NTS1R (18, 19). A recent complex structure indicated that SBI-553 is bound to a binding site at the interface between GRK2 and NTSR1, where it can enhance GRK2 binding and phosphorylation of receptor (19). In detail, SBI-553 forms predominately hydrophobic contacts with the intracellular residues from TM2, TM3, TM5, TM6, TM7, and H8 in NTS1R, as well as direct interactions with intracellular effectors (18, 19).

Some endogenous molecules can reportedly behave as allosteric modulators (110, 111). For example, certain bile acid-derivative cholic acids (e.g., deoxycholic acid (DCA), taurocholic acid (TCA), and taurodeoxycholic acid (TDCA) act as PAMs by binding an allosteric pocket formed by TM3, TM4, TM5, and ICL2 in G protein-coupled bile acid receptor (GPBAR) (110) (**Figure 2H**). In addition, certain endogenous lipids in membrane bilayers, such as phospholipid and cholesterol, can regulate signaling transduction by GPCRs (112–116). Cholesterol is an essential component of eukaryotic membranes and plays an important role in GPCR function and pharmacology (112, 117). Approximately 44% of human class A receptors are predicted to have a cholesterol binding site (118, 119). Indeed, high-resolution GPCR structures confirm the presence of lipid-binding sites in GPCRs. For example, a cholesterol molecule was found to bind to the cleft between TM1 and TM7 in the serotonin 1A receptor (5HT_1A_R), shaping the orthosteric ligand binding pocket by allosteric communication (111, 120) (**Figure 2I**). On the contrary, for β2AR, cholesterol was found to bind the surface of TM1, TM2, TM3 and TM4 and act as a NAM, since the bound cholesterol can increase the affinity for partial inverse agonist timolol and inhibit signaling pathway (118, 119).

## Structural basis for Class B GPCR allostery

Class B GPCRs are a small subfamily of 15 receptors, typically with a large N-terminal domain involved in recognition of peptide hormones (77, 121, 122). A prototypical example is glucagon-like peptide-1 receptor (GLP-1R), which predominately couples to the Gs effector and serves as an important drug target for the treatment of type 2 diabetes (123, 124).

**Figure 3:**
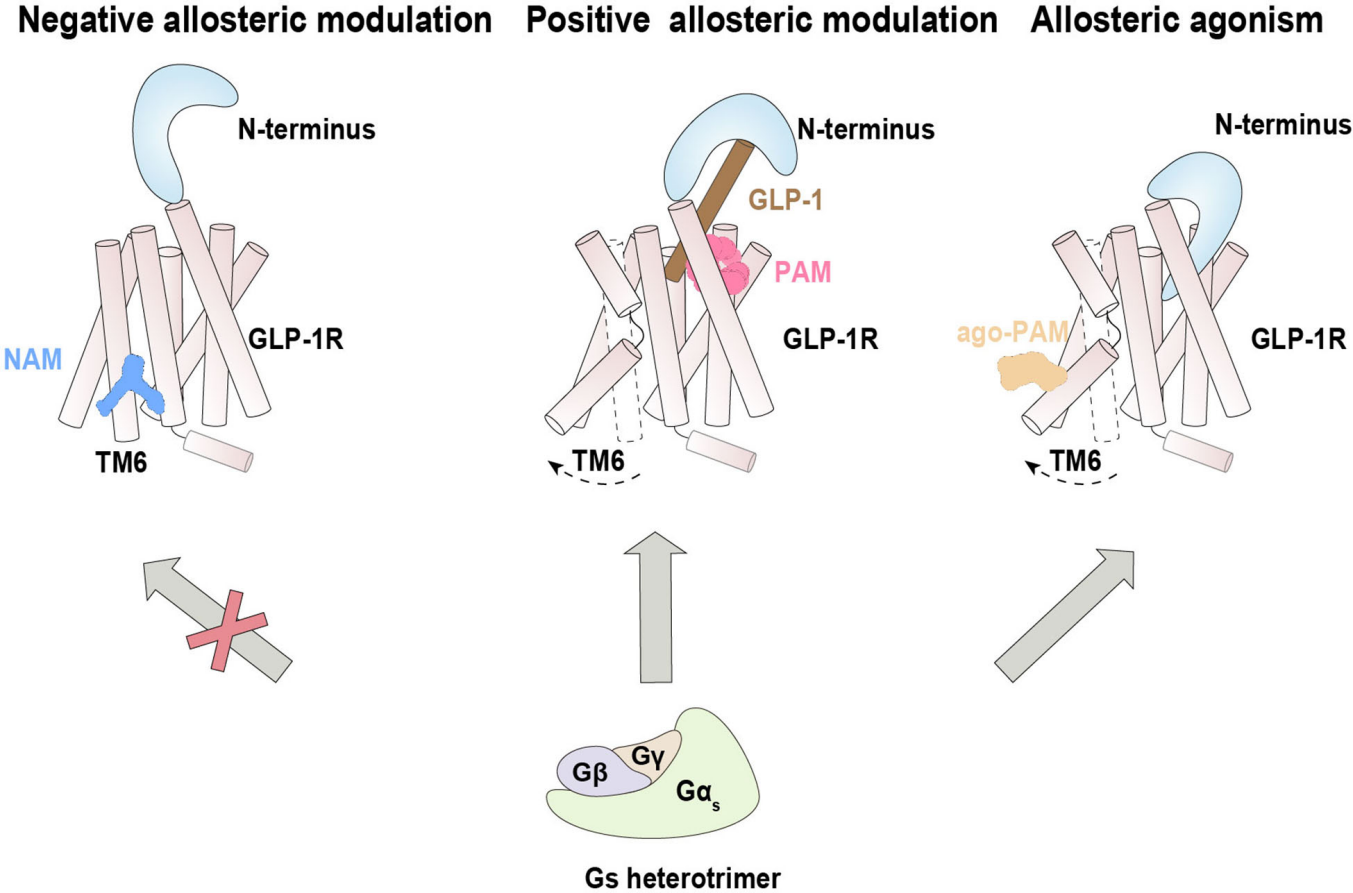
Allosteric modulation of GLP-1R signaling. Negative allosteric modulation of GLP-1R signaling (left), positive allosteric modulation of GLP-1R signaling (middle) and positive allosteric agonism of GLP-1R signaling (right).

PF-06372222 and NNC0640, two reported NAMs of GLP-1 with distinct scaffolds, bind a common extrahelical binding pocket formed by TM5–TM7 near the intracellular part (72). These NAMs inhibit the Gs protein pathway by restricting the outward movement of TM6 from the inactive state, which is crucial for receptor activation. Coincidentally, previous studies revealed that NNC0640 and MK-0893 (another NAM for the glucagon receptor) also bind in this region, although the allosteric pocket is not fully conserved between these two receptors (75, 76) (**Figure 3**). These results suggest a common mechanism by which NAMs of Class B GPCRs inhibit Gs protein coupling by preventing the conformational transition of TM6 to an active state, although the allosteric antagonist CP-376395 of CRF_1_R were identified to bind within the helical bundle of TM3, TM5 and TM7 (77).

LSN3160440 was characterized as a PAM of GLP-1R that enhances both the efficacy and potency of G protein signaling. Structural determination suggests that binding of LSN3160440 within the transmembrane helical bundle near TM1 and TM2 simultaneously interacts with the orthosteric ligand GLP-1(9-36) and GLP-1R (73) (**Figure 3**). This unique binding mode appears to stabilize the interacting interface between the orthosteric agonist and receptor, thereby enhancing the binding affinity of GLP-1(9-36) and elevating potential receptor activation.

Spatially distinct from LSN3160440, the ago-PAM compound 2 was found to covalently bond to the C347^6.36b^ residue located on the intracellular side of TM6 of GLIP-1R (74). Intriguingly, in a reported structure of compound 2/GLP-1/GLP-1R/Gs, compound 2 seemed to remotely induce insertion of the N-terminal domain into the orthosteric binding pocket, thus triggering activation of G1P-1R underlying its agonistic property (**Figure 3**). In addition, compound 2 cooperated with diverse orthosteric agonists to positively modulate cAMP signaling (125), enhanced the binding ability of agonists, and strengthened the G protein-receptor interface (74).

Together, the structural discovery of allosteric sites expands our understanding of negative and positive allosteric modulation of downstream signaling of Class B GPCRs.

## Structural basis for Class C GPCR allostery

**Figure 4:**
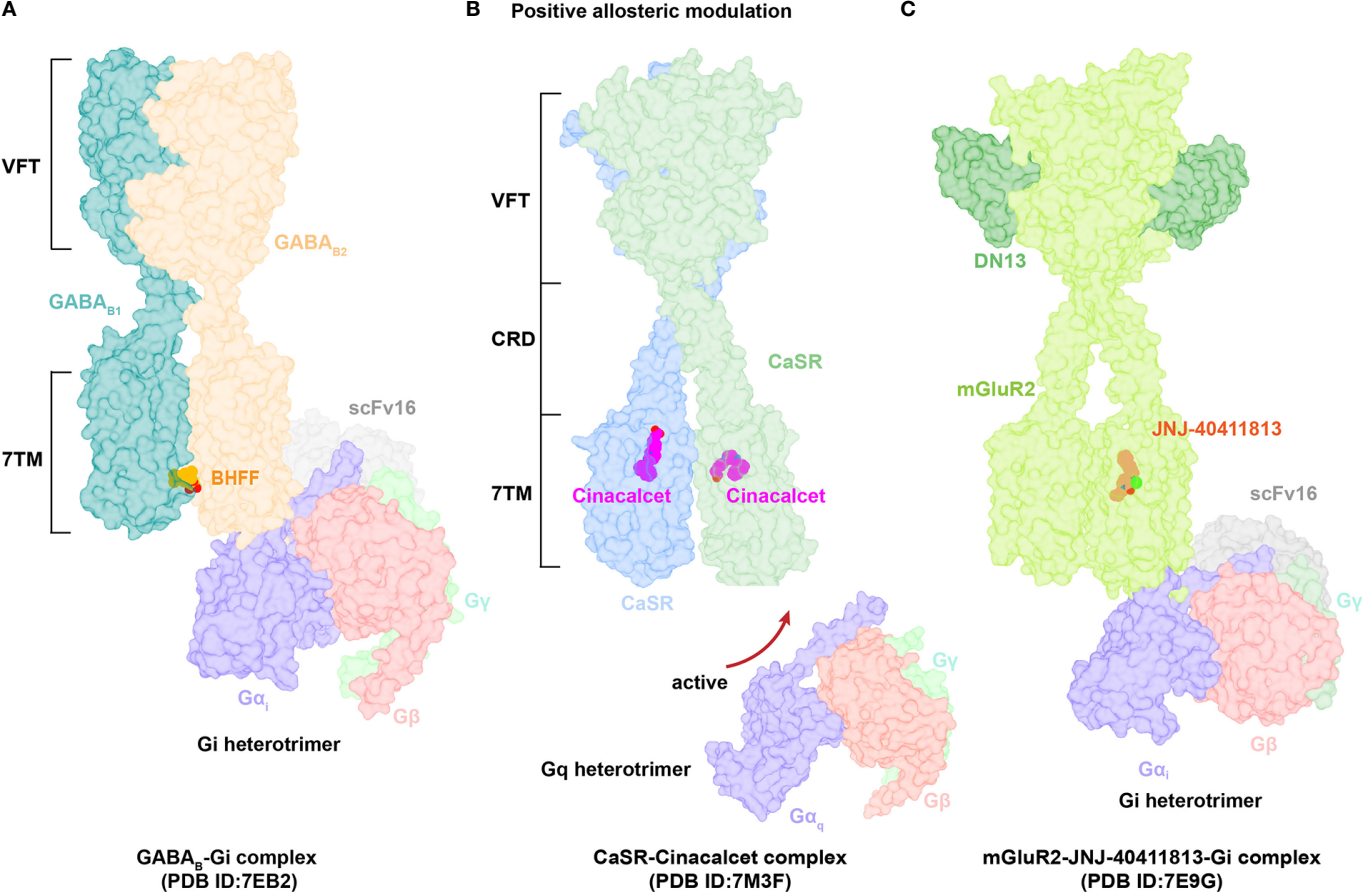
Structural basis of allosteric regulation in Class C GPCRs. **(A)** Cryo-EM structural model of active-state GABA_B_ complexed with PAM BHFF (orange). **(B)** Cryo-EM structural model of active-state CaSR complexed with PAM cinacalcet (magentas). **(C)** Cryo-EM structural model of active-state mGluR2 complexed with PAM JNJ-40411813 (salmon).

Class C GPCRs mainly include γ-aminobutyric acid B (GABA_B_) receptors, CaSR, and metabotropic glutamate (mGlu) receptors, which are very important therapeutic targets for the treatment of CNS disorders (126). Class C receptors function in a dimer state (either hetero or homo), each with three domains: Venus flytrap (VFT), a cysteine-rich domain (CRD, except GABA_B_ receptors), and a seven-transmembrane-helices domain (TMD) (127) (**Figure 4**).

**Figure 5:**
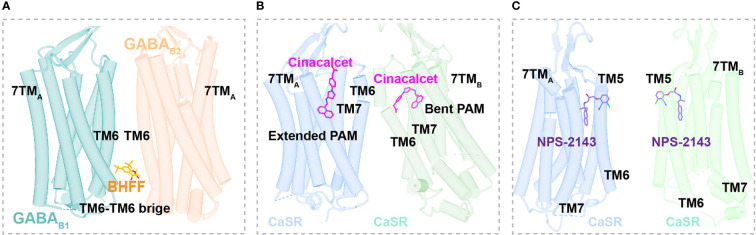
Binding Sites of allosteric modulators in Class C GPCRs. **(A)** Close view of binding sites of positive allosteric modulator BHFF (orange, sticks) in GABA_B_ (dark cyan and light salmon, cartoon) (PDB ID: 7EB2). **(B)** Close view of binding sites of positive allosteric modulator cinacalcet (magentas, sticks) in CaSR (cornflower blue and medium aquamarine, cartoon) (PDB ID: 7M3F). **(C)** Close view of binding sites of negative allosteric modulator NPS-2143 (medium purple, sticks) in CaSR (cornflower blue and medium aquamarine, cartoon) (PDB ID: 7M3J).

The GABA_B_ heterodimer includes two subunits: GABA_B1_ (responsible for endogenous ligand binding) and GABA_B2_ (responsible for Gi/o protein activation) (128). Several compounds reportedly act as PAMs of GABA_B_ receptors, such as CGP7930 (129) (the first characterized PAM of GABA_B_ receptors), *R*,*S*-5,7-di-tert-butyl-3-hydroxy-3-trifluoromethyl-3*H*-benzofuran-2-one (BHFF) (130), and GS39783 (131). Notably, they all exert positive allosteric effects on both ligand binding and the signaling response of GABA (78). A reported structure of the GABA_B_-Gi signaling complex bound to BHFF and an agonist reveals that the PAM links TM6 from both receptor subunits, forming a TM6-TM6 bridge required for receptor activation (81) (**Figures 4A**, **5A**). In contrast, the first identified NAM of the GABA_B_ receptor, CLH304a, can attenuate intracellular signaling (132).

Cinacalcet, the first GPCR allosteric drug approved by the FDA (21, 22), acts as a PAM of CaSR (25). Two other PAMs, evocalcet and etelcalcetide, have been investigated in clinical trials to treat secondary hyperparathyroidism and familial hypocalciuric hypercalcemia type 1 (FHH1) (83). Cinacalcet adopts two different binding conformations that allow it to bend into the seven-transmembrane core of each subunit of CaSR; purportedly, the extended version stabilizes the active state to promote G protein activation (**Figures 4B**, **5B**). In addition, L-amino acids can bind to one VFT cleft of CaSR to increase its sensitivity to fast fluctuations of Ca^2+^ concentrations. Compared with the asymmetric activation of CaSR induced by a PAM, the TMD of CaSR bound to the NAM NPS-2143 is absolutely symmetrical (83) (**Figure 5C**).

mGluRs can be divided into three groups: Group I (mGluR1 and mGluR5) couples to Gq/G11 proteins and activates phospholipase Cβ, resulting in production of inositol 1,4,5-trisphosphate (IP3) and diacylglycerol; Group II (mGluR2 and mGluR3) and Group III (mGluR4, mGluR6–mGluR8) predominantly couple to Gi/o, inhibiting adenylyl cyclase and cAMP production (127, 133). The ligand FTIM was characterized as a NAM of mGluR1; its recognition pocket is constituted by residues from extracellular loop 2, TM2–TM3, and TM5–TM7 (85). Interestingly, VU0424465 (a PAM of mGluR5) could make TMD closer in the absence of an endogenous agonist, further triggering signal transduction (134). Mavoglurant acts as a NAM of mGlu5, which is used to treat fragile X syndrome. In comparison to the position of FITM in mGlu1, mavoglurant is found lower in the mGlu5 allosteric site (89). The ligand JNJ-40411813, a reported PAM of mGluR2, binds to one of the TMD required for Gi protein coupling to potentiate downstream signaling (38) (**Figure 4C**). Upon binding of the NAM VU0650786 to mGluR3, the TMD undergoes structural rearrangements to reduce the distance between TM3 and TM4 helices, subsequently decreasing cAMP inhibition. These findings confirm that VU0650786 stabilizes the inactive state of mGluR3 (88).

## Conclusion and perspectives

In this review, we summarized recent structural studies of allosteric regulation of GPCRs. Progress in defining GPCR structures has facilitated understanding of the complex pharmacological features of their allosteric modulation, providing structural clues for ligand optimization and design of novel allosteric therapeutics. Recent studies demonstrate the analgesic efficacy of allosteric modulators of A_1_R in rats with neuropathic pain (61), whereas SBI-553 (an arrestin-biased allosteric modulator of neurotensin receptor 1) shows efficacy in animal models of psychostimulant abuse (18). Thus, understanding how allosteric modulators bias mechanisms of GPCRs has the potential to improve the precision of treatments for various diseases, while structure-based allosteric agent discovery could accelerate translational studies of GPCR allostery. Nevertheless, some challenges remain to untap the mechanisms of GPCRs, namely: (i) more GPCR structures in complex with allosteric modulators are urgently needed to identify and characterize allosteric sites (e.g., no structure of a GPCR in complex with a biased allosteric modulator has been reported); (ii) divergent cooperative mechanisms of allosteric modulators with orthosteric ligands remain largely elusive; and (iii) recently NMR study of the PAM LY2119620 at the M2R (63) as well as single molecule FRET studies (134–137) that explore conformational changes at the mGlu2 in response to PAMs have begun to provide dynamic structural information for understanding the mechanism of GPCR allostery. Further exploration of the dynamic states of GPCRs in response to different types of ligands using biophysical techniques (e.g., nuclear magnetic resonance (NMR), electron paramagnetic resonance (EPR), hydrogen-deuterium exchange (HDX), and time-resolved single molecules) will facilitate additional identification of intermediate receptor states in response to allosteric modulators. In sum, the structural determination of GPCRs in complexes with allosteric modulators will improve our understanding of receptor allostery.

## Author contributions

SiS, CZ, CW, and ZS discussed and formulated the focus of the review. SiS, CZ, CW, SuS, and ZL conducted the literature search and drafted the manuscript under the supervision of ZS and WY. ZS and SiS evaluated and revised the manuscript for final submission. All authors contributed to the article and approved the submitted version. 
